# Optimised Pre-Analytical Methods Improve *KRAS* Mutation Detection in Circulating Tumour DNA (ctDNA) from Patients with Non-Small Cell Lung Cancer (NSCLC)

**DOI:** 10.1371/journal.pone.0150197

**Published:** 2016-02-26

**Authors:** James L. Sherwood, Claire Corcoran, Helen Brown, Alan D. Sharpe, Milena Musilova, Alexander Kohlmann

**Affiliations:** Personalised Healthcare & Biomarkers, Innovative Medicines and Early Development Biotech Unit, AstraZeneca, Darwin Building, 310 Cambridge Science Park, Milton Road, Cambridge, CB4 0WG, United Kingdom; University of Navarra, SPAIN

## Abstract

**Introduction:**

Non-invasive mutation testing using circulating tumour DNA (ctDNA) is an attractive premise. This could enable patients without available tumour sample to access more treatment options.

**Materials & Methods:**

Peripheral blood and matched tumours were analysed from 45 NSCLC patients. We investigated the impact of pre-analytical variables on DNA yield and/or *KRAS* mutation detection: sample collection tube type, incubation time, centrifugation steps, plasma input volume and DNA extraction kits.

**Results:**

2 hr incubation time and double plasma centrifugation (2000 x g) reduced overall DNA yield resulting in lowered levels of contaminating genomic DNA (gDNA). Reduced “contamination” and increased *KRAS* mutation detection was observed using cell-free DNA Blood Collection Tubes (cfDNA BCT) (Streck), after 72 hrs following blood draw compared to EDTA tubes. Plasma input volume and use of different DNA extraction kits impacted DNA yield.

**Conclusion:**

This study demonstrated that successful ctDNA recovery for mutation detection in NSCLC is dependent on pre-analytical steps. Development of standardised methods for the detection of *KRAS* mutations from ctDNA specimens is recommended to minimise the impact of pre-analytical steps on mutation detection rates. Where rapid sample processing is not possible the use of cfDNA BCT tubes would be advantageous.

## Introduction

The need for accurate mutation detection from Non-Small Cell Lung Cancer (NSCLC) tumour tissue has become established, along with the need to identify potential responders to personalized medicines such as tyrosine kinase inhibitors (TKIs); e.g. *EGFR* TKIs, erlotinib, gefitinib and the T790M directed TKI osimertinib)[1]. However, evaluable tumour tissue is not always available for NSCLC patients[2]. For example, in the recent Iressa Follow Up Measure (IFUM) study, tumour mutation status was unable to be determined in 19% of eligible patients[3]. In the UK only 71% of lung cancer patients have a histological confirmation of their disease[4].

Due to the lack of availability of suitable tissue samples, circulating tumour DNA (ctDNA) is becoming increasingly important as an alternative source for detection of actionable mutations[5]. There will be a greater demand for biomarker testing on samples particularly in lung cancer where more targeted drugs are in development[6] such as the MEK1/2 inhibitors; selumetinib[7], cobimetinib (GDC-0973, XL-518) and trametinib and the T790M directed EGFR TKIs, osimertinib (AZD9291)[8] and rociletinib (CO-1686)[9]. Mutation testing of plasma also offers a minimally invasive option to characterize metastatic and/or resistant disease mechanisms when tissue or re-biopsy or unavailable.

The clinical utility of the detection of TKI sensitizing *EGFR* mutations in ctDNA has been proven when treating patients with gefitinib[3] in NSCLC and with erlotinib in colorectal cancer (CRC)[10]. Screening for *KRAS* mutations in CRC and pancreatic cancer using ctDNA has also been explored along with *BRAF* in melanoma samples[11–14]. ctDNA is typically degraded to a length of 166 base pairs, most likely due to the nucleolytic processes that occur during apoptosis[15,16]. There is some evidence to suggest the ctDNA whilst in circulation has a half-life between 16 minutes[17] and around 2 hours[18,19] but with significant variation between cases. This is likely due to blood borne nuclease activity which degrades the fragments[20] in 10 base pair intervals[18] and the liver also clears fragments from the circulation[21]. Detection of mutations in ctDNA is difficult due to the low amount of mutant alleles in a background of wild-type DNA. It is present at very low levels (<1%)[22] and can vary from patient to patient due to complex biological processes such as gene amplification seen with *EGFR*[23]. Sensitive methods are imperative to give reliable results. Pre-analytic variables such as peripheral blood collection, processing, shipment and storage methods can affect detection rates.

Currently tumour tissue remains the recommended gold standard sample type due to the relatively low detection rate in ctDNA when compared to tumour tissue[3,24]. Detection of ctDNA ranges from 62% to 65.7% sensitivity for *EGFR* mutations in NSCLC[3,25] and from 25% to 41% sensitivity for *KRAS* mutations in CRC[26,27].

One of the greatest challenges in the detection of actionable mutations in ctDNA is the instability of white blood cells after collection. White blood cells break down post blood draw, leading to an increase in gDNA (genomic DNA)[28,29]. The increased abundance of normal bystander gDNA dilutes the tumour derived ctDNA making it more difficult to detect actionable mutations[30] and demands that techniques more sensitive than those used for mutation detection in tissue samples be used. Previous studies have investigated the use of improvement methods but in limited sample numbers[30–32]. Current recommendations for assessing ctDNA include: plasma rather than serum samples, use of EDTA or cell-free DNA collection tubes with processing within 4 hr, double centrifugation and no more than three freeze thaw cycles of plasma specimens[31].

The purpose of this study was to investigate various methods to improve mutation detection in ctDNA from a standard 10 mL patient blood draw. We specifically investigated the use of different blood collection tubes. Using plasma from 45 NSCLC patients with matching tumour blocks we also assessed the impact on DNA yield and/or *KRAS* detection under the following conditions: 1) incubation time at room temp prior to plasma isolation; 2) single or double centrifugation; 3) plasma input volume and 4) various DNA extraction kits.

## Materials and Methods

### Ethics Statement

Ethics committee approval was not required in the instance of this specific work as the samples were collected commercially from the Manchester Cancer Research Centre (MCRC) Biobank, UK or Asterand Bioscience Royston, UK which have generic ethics approval: http://www.asterandbio.com/company/ethics/. Full written informed consent was obtained for all samples used in this study.

The MCRC Biobank is licensed by the Human Tissue Authority (licence number: 30004) and has been ethically approved as a research tissue bank by the South Manchester Research Ethics Committee (Ref: 07/H1003/161+5). A suite of standard Patient Information Sheets and Patient Consent Forms have been developed and approved and informed patient consent will always be obtained before patient samples are taken. The Biobank holds what is known as generic ethics approval.

This confers approval to anyone using samples from the MCRC Biobank, so researchers do not need to obtain their own ethical approval for relevant projects using Biobank samples: http://www.mcrc.manchester.ac.uk/Biobank/Ethics-and-Licensing.

All procedures were carried out in accordance with the Helsinki Declaration (1964, amended in 1975, 1983, 1989, 1996 and 2000) of the World Medical Association and all patient samples submitted for analysis were done so with the full informed consent of the patients.

All clinical data and samples were received anonymously.

### Patients

**Fig 1:**
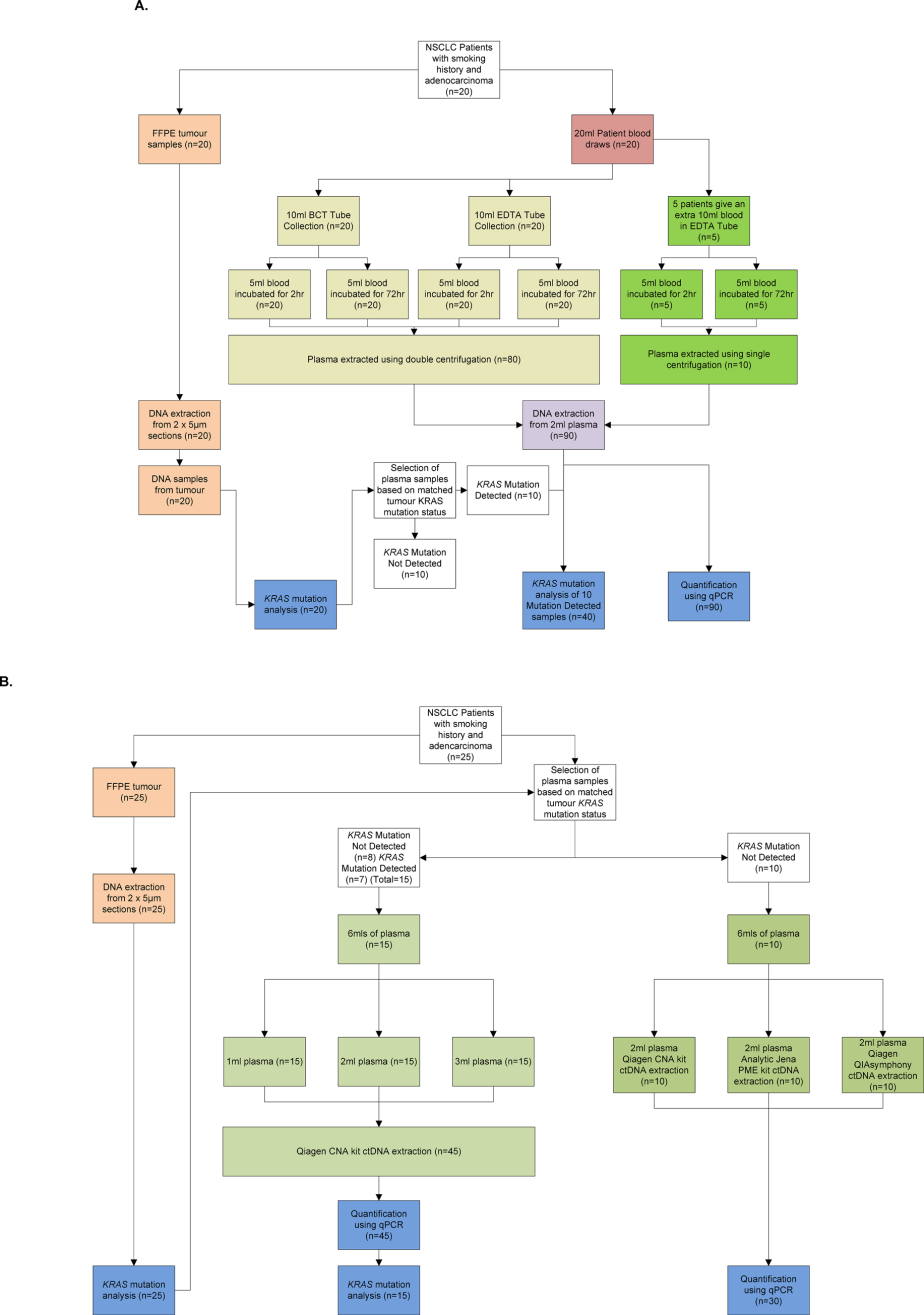
Sample workflow schematic. Diagram showing the experimental workflow for the different sample cohorts. Panel A. Comparison of Streck Cell Free (BCT) and EDTA tubes in addition to single and double centrifugation on DNA yield and mutation detection. B. Comparison of different plasma volumes and ctDNA extraction kits on DNA yield and mutation detection.

Two collections of NSCLC samples consisting of formalin-fixed paraffin-embedded (FFPE) tissue and matched plasma were used in this study (Fig 1). For the assessment of different plasma stabilisation methods, a collection of matched plasma and FFPE tissue weighted to Caucasian patients with adenocarcinoma and with a smoking history was used (n = 20; MCRC Biobank collaborating NHS Trusts). Matched plasma and FFPE tissue were used for comparing different volumes of plasma (n = 15; Asterand Bioscience, Royston, UK) and comparing different plasma DNA extraction kits (n = 10; Asterand Bioscience).

### Blood collection, plasma isolation and storage

For the assessment of different plasma stabilisation methods, 10 mL peripheral blood was collected in an EDTA blood collection tube (Vacutainer K2EDTA) (Becton, Dickinson, Oxford, UK) and 10 mL in a Cell-Free DNA™ BCT (cfDNA BCT) (Streck, Omaha, NE) for 20 patients, according to the instructions for use (inverting 10 times). Each 10 mL volume was then split into 2 x 5 mL aliquots and then incubated at room temperature for either 2 hr or 72 hr. Plasma was then isolated from the blood sample by performing a 2000 x g centrifugation for 10 min and carefully removing the plasma. The removed plasma was then centrifuged again at 2000 x g for 10 min before removal of the supernatant plasma for ctDNA extraction. For 5 of the 20 patients an additional 10 mL blood draw was collected in an EDTA tube and split into 2 x 5 mL volumes. The blood was then incubated at room temperature for either 2 hr or 72 hr prior to a solitary centrifuge spin at 2000 x g to isolate plasma (Fig 1A). For long-term storage, all plasma was stored at -80°C in 1 mL aliquots prior to DNA extraction.

For comparison of different plasma volumes and different DNA extraction kits, blood was collected in EDTA tubes and centrifuged once for 10 min at 1300 x g. Plasma was harvested in 1 mL aliquots and stored at -80°C within 1 hr of blood collection. Aliquots from the same patient were thawed on ice and pooled per patient to generate >6 mL total. Pooled plasma was then mixed thoroughly and dispensed into 1, 2 and 3 mL volumes for the plasma volume comparison study (n = 15), and 3 x 2 mL aliquots for the DNA extraction kit comparison study (n = 10) (Fig 1B). All plasma samples were then frozen at -80°C.

All tissue collections were performed concurrently although those collected by Asterand Bioscience were conducted under the limitations of availability of such matched sample sets, therefore some collection parameters were not ideal for cfDNA e.g. collection with one centrifugation step as above.

### DNA extraction from plasma

For comparison of blood stabilisation and plasma volume the QIAamp Circulating Nucleic Acid Kit (Qiagen, Hilden, Germany) was used. In addition, a comparison of three DNA extraction kits was performed on equal volumes of plasma (2 mL each) (Fig 1B). The following kits were used: PME free-circulating DNA Extraction Kit protocol (Analytik Jena, Jena, Germany) for 2–5 mL extractions using lysis solution GS/Binding solution VL system and DSP Virus/Pathogen Midi Kit performed on QIAsymphony (Qiagen) and the QIAamp Circulating Nucleic Acid Kit (QIAGEN). QIAsymphony extractions were performed at the Qiagen applications lab (Hilden). All plasma samples were processed according to the manufacturers’ protocols with the exception of the PME kit where an initial 20 min incubation instead of 10 min was performed and the plasma centrifugation steps were carried out at 3750 x g vs 4500 x g. All DNA was eluted in 80 μL within the range specified by the kit.

### DNA extraction from FFPE tissue

DNA was extracted from 2 x 5 μm freshly-cut FFPE tissue sections according to the manufacturer’s protocol using the QIAamp DNA FFPE Tissue Kit (Qiagen) and eluted in 100 μL of buffer.

### DNA quantification

All eluted DNA was mixed thoroughly (vortexed) prior to measurement by quantitative PCR (qPCR) which was carried out using the ABI TaqMan® RNase P Detection Reagents Kit and TaqMan® Universal PCR Master Mix (Thermo Fisher Scientific, Waltham, MA, USA) on a Quantstudio Dx instrument (Life Technologies, CA. USA). Reactions were prepared using 10 μL of Master Mix, 1 μL of Assay Mix, 5 μL of DNA and 4 μL of Nuclease free water (Qiagen, Hilden, Germany). Cycling conditions were 95°C for 10 minutes then 94°C for 15 seconds and 60°C for 1 minute cycled 40x. A series of 10 serial dilutions of Human Genomic DNA (Roche Diagnostics, Mannheim, Germany) from 100 ng/μL to 0.195 ng/μL was used in duplicate to produce the standard curve. The amplicon size of the RNase P assay was 87 base pairs, which is appropriate for use on ctDNA.

### *KRAS* testing

*KRAS* mutation status was characterised from FFPE tissue-derived DNA using the *therascreen KRAS* RGQ PCR kit (Qiagen) according to the manufacturer’s protocol. The therascreen kit employs amplification refractory mutation system (ARMS) PCR technology combined with the Scorpions detection technology[33]. The mutation status of a given sample is established by comparison of the specific *KRAS* mutation assay against a control assay in *KRAS* exon 4 to generate a change in Cycle Threshold (∆CT). The ∆CT is the obtained by subtracting the control CT from the mutation assay CT. The ∆CT is then compared to the validated cut off criteria for the ∆CT in order to establish mutation status. *KRAS* mutation detection was performed in the same way on ctDNA samples from patients with *KRAS* mutations detected in the matching FFPE tissue-derived DNA. Samples processed by different pre-analytical methods were assayed in the same RotorGeneQ (RGQ) run. Plasma samples were only assessed for the known *KRAS* mutation as obtained in the matched tissue.

### Statistical analysis

Statistical analysis was performed using paired Student’s *t*-test in Microsoft Excel where p<0.05 was deemed significant. Linear regression analysis and the calculation of R^2^ were performed on GraphPad Prism (Version 6.01) and p-values were calculated based on deviation from zero. Graphs were generated in GraphPad Prism.

## Results

### Quantification of DNA following different plasma processing steps

**Fig 2:**
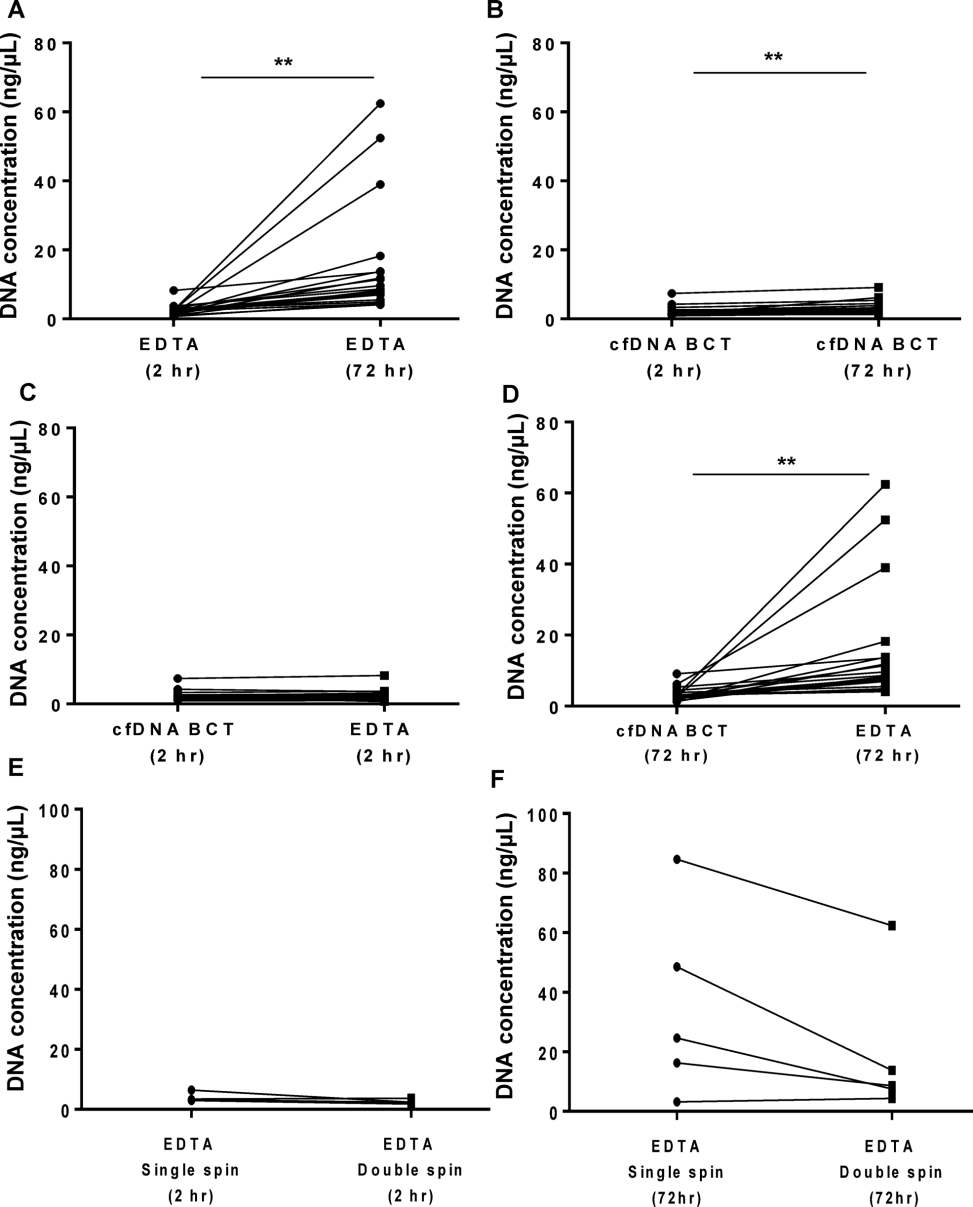
Plasma processing method comparison. Whole blood was collected from 20 NSCLC patients and processed using various tube types, incubation times and centrifugation steps. A subset of these patients (n = 5) had whole blood processed to assess a single vs. double centrifugation step. DNA was measured using the ABI TaqMan® RNase P Detection Reagent Kit **A:** Blood was collected using EDTA blood collection tube (EDTA) and processed after 2 hr or 72 hr. **B:** Blood was collected using cell free DNA blood collection tube (cfDNA BCT) and processed after 2 hr or 72 hr. **C:** Blood was collected using EDTA or cfDNA BCT tubes and processed after 2 hr. **D:** Blood was collected using EDTA or cfDNA BCT tubes and processed after 72 hr. **E:** Blood collected in EDTA tubes after 2 hr incubation and processed using either single or double centrifugation. **F:** Blood collected in EDTA tubes after 72 hr incubation and processed using either single or double centrifugation. Results are displayed for each patient. Statistical analysis was performed using a paired Student’s t-test where; **p<0.01.

To understand whether the methods of plasma processing may influence the levels of contaminating gDNA present, we collected blood from 20 NSCLC patients and processed the plasma under various conditions (Fig 1A and Fig 2). EDTA collection tubes and longer incubation time was significantly (p<0.01) associated with increased DNA yield, mean±SD: EDTA tube/ 2 hr incubation (2.49±1.60 ng/μL) versus EDTA tube/ 72 hr incubation (15.12±16.44 ng/μL) (Fig 2A). A low but significant (p<0.01) increase in DNA yield was also observed with cell-free DNA Blood Collection tubes (cfDNA BCT) at 72 hr (3.31±1.82 ng/μL) compared with cfDNA BCT/ 2 hr (2.39±1.42 ng/μL) (Fig 2B). There was no significant difference in total DNA yields observed when plasma was processed within 2 hr using either tube type (Fig 2C). The DNA yield from plasma processed after 72 hr using cfDNA BCT tubes was significantly (p<0.01) lower than plasma processed using EDTA tubes (Fig 2D)."

A subset of these patient samples (n = 5) was further evaluated for the influence of single or a double centrifugation step on DNA yields (Fig 2E and 2F). When processed within 2 hr, centrifuging the plasma samples twice did not have a significant effect on DNA yield compared to single spin (Fig 2E). However, when processed after 72 hr, a double centrifugation step resulted in decreased mean DNA yield (19.35±24.3 ng/μL) compared to single centrifugation processing (35.47±32.1 ng/μL). A reduction in yield was observed in 4 out of the 5 samples analysed (Fig 2F) however this was not statistically significant (p = 0.058). Taken together, these results demonstrate that the levels of DNA yields can vary depending on the method by which plasma is processed with non-optimal methods.

### *KRAS* mutation detection following different plasma processing steps

Application of the various plasma processing methods was also investigated for *KRAS* mutation detection in 20 patient samples (Fig 1A). Prior to performing *KRAS* mutation detection on any plasma derived DNA, the corresponding matched FFPE tissue was screened using the *therascreen KRAS* RGQ PCR kit to identify patients with mutation positive tumours (n = 10/20; data not shown). In this cohort, 50% (n = 5) had *KRAS* mutations identified in the matched plasma processed in cfDNA BCT collection tubes within 2 hr and 40% (n = 4) at 72 hr. However, this detection rate decreased when compared to a considered non-optimal method, e.g. EDTA collection tubes after 2hr, 40% (n = 4) of mutations were detected dropping to 20% (n = 2) after 72 hrs (Table 1) in the same samples. Individual (CT) values, total amplifiable KRAS DNA (KRAS control) and ΔCT values for all 10 plasma samples are displayed in S1 Table. It should be noted that *KRAS* mutation status was established using the Qiagen therascreen *KRAS* RGQ PCR handbook, ΔCT cut off values range from 6.6 to 8 for the mutations detected. Due to the low number of samples with *KRAS* mutations that were detectable in ctDNA, it is not possible to attach statistical significance.

**Table 1:** *KRAS* mutation detection on plasma from NSCLC patients.

	Patient 2	Patient 6	Patient 9	Patient 12	Patient 19
Mutation:	p.Gly12Asp	p.Gly12Asp	p.Gly12Arg	p.Gly12Val	p.Gly12Val
cfDNA BCT + double spin after 2 hr	**Mutation Detected**	**Mutation Detected**	**Mutation Detected**	**Mutation Detected**	**Mutation Detected**
cfDNA BCT + double spin after 72 hr	**Mutation Detected**	**Mutation Detected**	Mutation not detected	**Mutation Detected**	**Mutation Detected**
EDTA + double spin after 2 hr	**Mutation Detected**	**Mutation Detected**	Mutation not detected	**Mutation Detected**	**Mutation Detected**
EDTA + double spin after 72 hr	**Mutation Detected**	Mutation not detected	Mutation not detected	Mutation not detected	**Mutation Detected**

**Bold text** indicates “Mutation Detected” for easier reading.

Total amplifiable *KRAS* DNA (*KRAS* control) detection in all samples demonstrated a similar trend as observed for the DNA yield (qPCR) in Fig 2 (S1 Table). A decrease in CT value was demonstrated for non-optimal methods indicating greater contaminating DNA concentration; mean CT±SD: EDTA tube/2hr incubation (29.0±1.0), EDTA tube/72hr incubation (25.5±1.5), cfDNA BCT/2hr incubation (29.4±1.1), and cfDNA BCT/ 72 hr incubation (28.5±1.0), respectively (S1 Table). These results demonstrate that both *KRAS* mutation detection and also total amplifiable *KRAS* DNA can be influenced depending on the method of plasma processing used.

### Quantification of DNA following different plasma input

**Fig 3:**
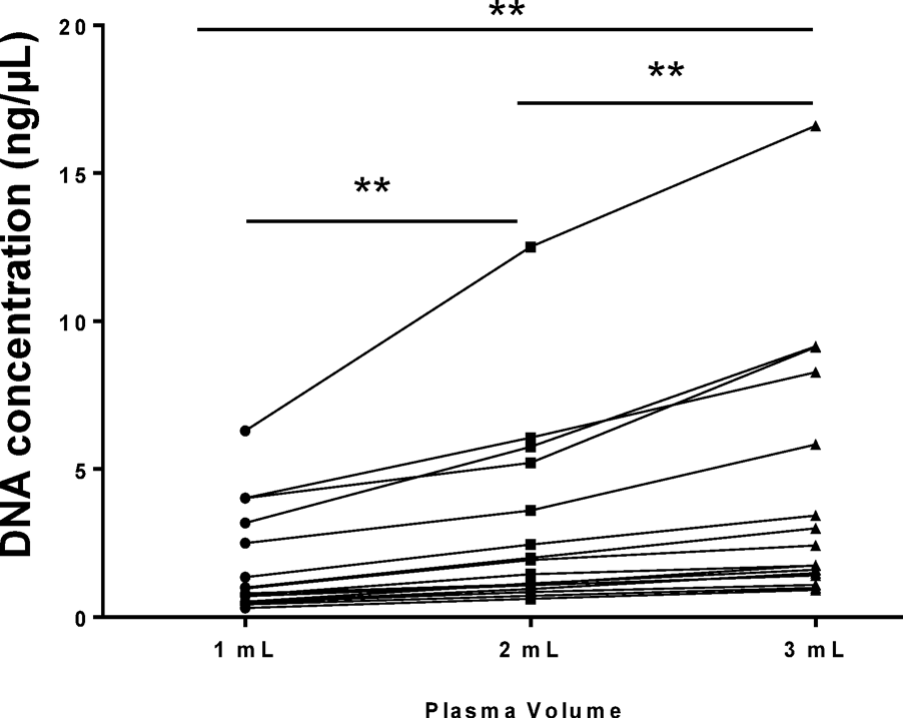
Plasma input volume comparison. Three volumes of plasma (1 mL, 2 mL and 3 mL) were processed from each sample in a cohort of 15 NSCLC patients. DNA was extracted using the QIAamp CNA Kit and was measured by qPCR using the ABI TaqMan® RNase P Detection Reagent Kit. Results are displayed for each patient. Statistical analysis was performed using a paired Student’s t-test where; **p<0.01.

As expected, a significant increase in DNA yield was observed with increasing sample volume (p<0.01) from 15 patients (Fig 3). An increase in yield will increase the concentration and the absolute copy numbers of ctDNA available for analysis thereby making mutation detection more likely. The mean DNA yield from 3 mL plasma was 4.39 ±4.55 ng/μL compared to 3.03±3.24 ng/μL (2 mL plasma) and 1.79±1.81 ng/μL (1 mL plasma). From these 15 patients, 7 had *KRAS* mutations in corresponding matched tissue In this cohort, one patient (number 23) had a detectable *KRAS* mutation in plasma for all three volumes the remaining patient samples having ΔCT values outside of the cut off criteria for the test (S2 Table). This may be due to this particular collection of matched plasma samples being drawn into ordinary EDTA tubes, with only a single spin reflecting the usual detection rate that arises in non-optimal methods[34]. Furthermore these specific samples were primarily derived from stage I or II NSCLC (13/20) which could contain lower levels of ctDNA from the tumour[35]. Interestingly, *KRAS* amplification control demonstrated a similar trend to the DNA yield (qPCR) in Fig 2. Lower mean CT values for the *KRAS* control were observed with increased plasma volume. The lower CT values demonstrated a significant increase (p<0.001) in *KRAS* control detection (mean CT±SD) for 3 mL plasma (26.5±1.7) compared to 2 mL plasma (27.1±1.8) and 1 mL plasma (28.1±1.7) samples (S2 Table). These data demonstrate that varied input plasma volume can impact on DNA yield and total amplifiable *KRAS*. For *KRAS* mutation detection, however, a larger cohort of detectable mutant samples is required for further analysis.

Whilst no conclusion can be made from one single sample, all samples increased in DNA yield as expected. The increased numbers of *KRAS* amplifiable DNA copies obtained through extraction of more plasma, could influence the detection rate in these samples if more sensitive methods, e.g. digital PCR were employed.

### DNA extraction kit comparison

**Fig 4:**
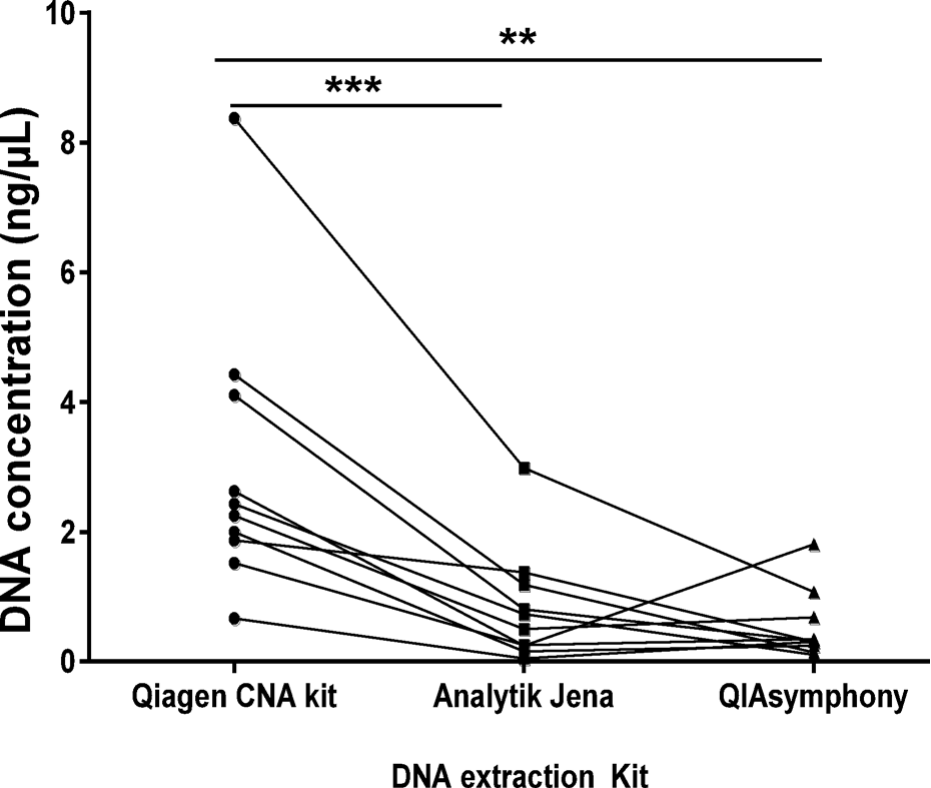
DNA extraction kit comparison. Equal volumes of plasma (2 mL) from 10 NSCLC patients were processed using three different DNA extraction methods: QIAamp Circulating Nucleic Acid Kit (Qiagen CNA Kit); PME free-circulating DNA Extraction Kit (Analytik Jena) and the DSP Virus/Pathogen Midi Kit performed on QIAsymphony (QIAsymphony). DNA was measured by qPCR using the ABI TaqMan® RNase P Detection Reagent Kit. Results are displayed for each patient. Statistical analysis was performed using a paired Student’s t-test where; **p<0.01, ***p<0.001.

Equal volumes (2 mL) of plasma from 10 patients were subjected to DNA extraction using three distinct methods. All three methods demonstrated successful extraction of DNA (Fig 4) with Qiagen’s QIAamp Circulating Nucleic Acid kit yielding the highest DNA yield, i.e. mean±SD (3.03±2.19ng/μL) compared to Analytic Jena’s PME free-circulating DNA Extraction Kit protocol (0.83±0.88ng/uL; p<0.001) and compared to Qiagen’s DSP Virus/Pathogen Midi Kit performed on QIAsymphony (0.53±0.53ng/μL; p<0.01). The manual kits are simple to use and suited to small to medium batch sizes whereas the QIAsymphony requires specific training but is more suited to high throughput and continuous processing.

## Discussion

Personalised therapy in cancer often relies on testing for DNA mutations using a companion diagnostic assay to identify patients that will more likely respond to targeted therapies. At present the most commonly used specimen for detection of actionable DNA mutation changes is tumour tissue, typically FFPE tissue. However, a tissue sample is not always available[3] either due to depletion of the diagnostic block for other molecular pathology investigations or because the patient has never been biopsied for other clinical reasons. Where tissue availability is an issue careful choice of highly parallel diagnostic platforms such as Next Generation Sequencing (NGS) or Matrix Assisted Laser Desorption/ Ionisation Time of Flight (MALDI-TOF), can delay depletion of the block[36].

Liquid biopsies (e.g. plasma) offer an alternative, temporal and less-invasive option for accurate mutation testing. Previous studies have shown that mutation testing of ctDNA has high specificity (>95%), but the sensitivity is lower (60%) compared to tissue[3,37]. Most of the documented evidence in NSCLC to date relates to *EGFR* testing. Some studies have investigated *KRAS* mutations from ctDNA in colorectal cancer patients [12,26,34].

Technologies and methods which can improve the isolation of ctDNA and reduce wild-type DNA contamination are emerging[31]: avoiding heparin tubes[38], use of plasma rather than serum[39] and avoidance of freezing samples[28]. These include step changes in plasma processing for example centrifugation steps (speed and number of spins)[40] and more sophisticated improvements such as a specialized collection tubes, i.e. cell free DNA blood collection tubes that stabilize the white cells in the blood [30,32]. Although the potential clinical utility of *KRAS* mutation detection from plasma has also been explored[41] in NSCLC, the effect of these processing modifications on *KRAS* mutation detection in NSCLC has not yet been specifically investigated.

The aim of this study was to identify optimal pre-analytical processing steps to obtain ctDNA from plasma from lung cancer patients. In this study we measured the impact of these steps on DNA yields and *KRAS* mutation detection rates.

Quantitative PCR (qPCR) was thought to be the most appropriate DNA quantification method as it characterises amplifiable DNA molecules only[28]. The method used employed an 87 base pair amplicon which would amplify ctDNA which is typically degraded to 166 base pairs(40). It should be noted however, that the use of other methods is appropriate when used as part of a previously validated method.

Circulating DNA exists naturally but is often elevated in cancer patients[19]. The quantity of ctDNA in a sample varies dependent on disease setting and stage[35]. The detection of mutations from ctDNA relies heavily on the stability of the sample. There has been an emergence of alternative blood collection tubes that stabilise blood cells, e.g. CellSave Preservative Tubes (Cell Search, South Ritan, NJ, USA) and cfDNA BCT (Streck). In this study we focussed on the assessment of cfDNA BCT tubes because these were the only commercially available product specifically designed to stabilise blood for ctDNA assessment. The stabilising solution contained in cfDNA BCT tubes does not affect downstream molecular analysis by PCR[42] as we also observed in this study. Previous studies have shown that cfDNA BCT tubes preserve blood enabling the detection of circulating free DNA using plasma from healthy donors[30,32] or plasma spiked with cancer cells [43]. However, to the best of our knowledge there are no studies that have used these tubes to assess DNA yield and specific mutations in plasma from NSCLC patients.

We here demonstrated that when using different processing methods such as a 72 hr incubation prior to processing, cfDNA BCT tubes were better at stabilising the blood compared to EDTA collection tubes as seen by a reduction in DNA yields. As such, when immediate processing (<2 hr) of plasma is not possible, cfDNA BCT tubes provide a superior alternative to standard EDTA collection tubes. This is particularly important in a clinical environment where blood is collected but is unable to be processed immediately.

It has been reported that an initial slow centrifugation (<1,600 x g for 10 min) followed by a second centrifugation, at high speed (<16,000 x g for 10 min) is necessary for isolating cell-free plasma[40,44] as seen by a decrease in DNA yield. Importantly, clinical laboratories do not always have the capability to perform a faster second centrifugation. Our study confirmed that gDNA contamination was lower from plasma isolated with a double centrifugation compared to a single centrifugation supporting previous observations. In these circumstances it is recommended that a second centrifugation at the lower speed is more efficient at removing cells than a single centrifugation[45]. Moreover, increasing the plasma volume, increased the DNA yield and lowered the *KRAS* control as expected[45].

There are a number of DNA extraction options that can be used for ctDNA analysis from plasma or serum (S3 Table). We chose to compare three methods, all specifically designed for extraction of circulating DNA, which enabled the use of 2 mL of input plasma. We selected one automated method (QIAsymphony), one commonly used method (QIAamp CNA kit) and one less documented method (Analytik Jena). While we observed that methods yielded sufficient DNA for downstream application however the yields differed significantly across the assays. The different yields as measured by qPCR may have resulted from isolation of fragments of differing size distribution due to size exclusion of nucleic acids in a given kit. Due to the variation in performance of commercial kits, a validated method should be used when analysing clinical samples. Therefore, careful consideration should be given when choosing an optimal ctDNA extraction method.

In conclusion, although individually some optimisation steps had a modest effect in this study, the accumulation of multiple modifications will ultimately lead to improved mutation detection rates in ctDNA. In particular, the inclusion of the cfDNA BCT tubes was shown to improve the stabilisation of peripheral blood compared to EDTA tubes after 72 hr incubation. Additionally, our study demonstrated that the number of centrifugation steps, input plasma volume and extraction kit used influence DNA yield. Further studies are warranted to establish if there is a benefit to patients by additional blood draw. When tumour tissue is not available, consideration of some of these optimal pre-analytical methods will improve the likelihood of successful mutation detection from plasma, further reducing the need for invasive tumour biopsies. Whilst previous studies have demonstrated that plasma processing has an impact on DNA yield, our study is the first to investigate the effect of this on lung cancer patient samples looking specifically in a *KRAS* mutation detection setting. The recommendations in this study could help improve NSCLC patient access to personalised medicines such as *EGFR* TKIs and also MEK inhibitors, if the clinical benefit of such drugs is proven.

## Supporting Information

S1 Table *KRAS* mutation detection details from ctDNA for 10 samples with *KRAS* mutations detected in matching tumour tissue following various plasma processing methods. (DOCX)

S2 Table *KRAS* mutation detection details from various input plasma volumes. (DOCX)

S3 Table List of commercially available cell free DNA extraction kits. (DOCX)
