# Supplementary material for: Optimised Pre-Analytical Methods Improve KRAS Mutation Detection in Circulating Tumour DNA (ctDNA) from Patients with Non-Small Cell Lung Cancer (NSCLC)
Source: PLoS One. 2016 Feb 26;11(2):e0150197. doi: 10.1371/journal.pone.0150197 (PMC4769175; doi:10.1371/journal.pone.0150197)
Supplement: S1 Table — (DOCX) [file pone.0150197.s001.docx]

| **S1 Table: *KRAS* mutation detection details from ctDNA for 10 samples with *KRAS* mutations detected in matching tumour tissue following various plasma processing methods*** | | | |  |
| --- | --- | --- | --- | --- |
| **Sample** | **Control CT** | **G12V** | **Delta CT** | ***KRAS* Status** |
| Patient 19 -a | 27.33 | 33.36 | 6.03 | Positive |
| Patient 19 -b | 26.39 | 31.6 | 5.21 | Positive |
| Patient 19 -c | 27.07 | 33.28 | 6.21 | Positive |
| Patient 19-d | 25.88 | 32.91 | 7.03 | Positive |
| **Sample** | **Control CT** | **G12R** | **Delta CT** |  |
| Patient 9 -a | 30.64 | 37.25 | 6.61 | Positive |
| Patient 9 -b | 30.38 | Not detected (>45CT) | N/A | Negative |
| Patient 9 -c | 30.96 | Not detected (>45CT) | N/A | Negative |
| Patient 9-d | 26.21 | Not detected (>45CT) | N/A | Negative |
| **Sample** | **Control CT** | **G12V** | **Delta CT** |  |
| Patient 12 -a | 28.8 | 36.12 | 7.32 | Positive |
| Patient 12 -b | 28.41 | 34.84 | 6.43 | Positive |
| Patient 12 -c | 28.95 | 35.1 | 6.15 | Positive |
| Patient 12 -d | 26.92 | 37.89 | 10.97 | Negative |
| **Sample** | **Control CT** | **G12D** | **Delta CT** |  |
| Patient 17 -a | 29.36 | 40.28 | 10.92 | Negative |
| Patient 17 -b | 28.92 | 40.59 | 11.67 | Negative |
| Patient 17 -c | 29.15 | Not detected (>45CT) | N/A | Negative |
| Patient 17 -d | 23.16 | 36.95 | 13.79 | Negative |
| **Sample** | **Control CT** | **G12C** | **Delta CT** |  |
| Patient 20 -a | 31.02 | Not detected (>45CT) | N/A | Negative |
| Patient 20 -b | 27.78 | Not detected (>45CT) | N/A | Negative |
| Patient 20 -c | 30.02 | Not detected (>45CT) | N/A | Negative |
| Patient 20 -d | 23.87 | 39.08 | 15.21 | Negative |
| **Sample** | **Control CT** | **G12D** | **Delta CT** |  |
| Patient 13 -a | 29.11 | 45.22 | 16.11 | Negative |
| Patient 13 -b | 28.56 | 43.87 | 15.31 | Negative |
| Patient 13 -c | 28.51 | 40.35 | 11.84 | Negative |
| Patient 13 -d | 26.2 | 37.14 | 10.94 | Negative |
| **Sample** | **Control CT** | **G12D** | **Delta CT** |  |
| Patient 14 -a | 29.02 | 44.27 | 15.25 | Negative |
| Patient 14 -b | 28.69 | 43.16 | 14.47 | Negative |
| Patient 14 -c | 29.09 | Not detected (>45CT) | N/A | Negative |
| Patient 14-d | 27.15 | 42.11 | 14.96 | Negative |
| **Sample** | **Control CT** | **G12D** | **Delta CT** |  |
| Patient 2 -a | 28.59 | 30.29 | 1.7 | Positive |
| Patient 2 -b | 28.39 | 29.94 | 1.55 | Positive |
| Patient 2 -c | 28.39 | 30.47 | 2.08 | Positive |
| Patient 2 -d | 25.71 | 30.15 | 4.44 | Positive |
| Patient 2 -e | 28.12 | 30.42 | 2.3 | Positive |
| Patient 2 -f | 23.61 | 29.88 | 6.27 | Positive |
| **Sample** | **Control CT** | **G12D** | **Delta CT** |  |
| Patient 6 -a | 30.29 | 33.66 | 3.37 | Positive |
| Patient 6 -b | 28.37 | 33.97 | 5.6 | Positive |
| Patient 6 -c | 28.59 | 33.76 | 5.17 | Positive |
| Patient 6 -d | 26.82 | 34.11 | 7.29 | Negative |
| Patient 6 -e | 28.6 | 33.99 | 5.39 | Positive |
| Patient 6 -f | 25.34 | 33.54 | 8.2 | Negative |
| **Sample** | **Control** | **G12C** | **Delta CT** |  |
| Patient 5 -a | 29.45 | Not detected (>45CT) | N/A | Negative |
| Patient 5 -b | 28.78 | Not detected (>45CT) | N/A | Negative |
| Patient 5 -c | 29.38 | Not detected (>45CT) | N/A | Negative |
| Patient 5 -d | 23.32 | 39.46 | 16.14 | Negative |
| Patient 5-e | 28.81 | Not detected (>45CT) | N/A | Negative |
| Patient 5 -f | 22.63 | 40.06 | 17.43 | Negative |
|  |  |  |  |  |
| **Key** |  |  |  |  |
| Sample letter | **Process step** |  |  |  |
| A | cfDNA BCT + double spin after 2hr | |  |  |
| B | cfDNA BCT + double spin after 72hr | |  |  |
| C | EDTA + double spin after 2hr | |  |  |
| D | EDTA + double spin after 72hr | |  |  |
| E | EDTA + single spin after 2hr | |  |  |
| F | EDTA + single spin after 72hr | |  |  |
| *Qiagen therascreen Delta Ct cutoff  G12V=7.5  G12R=8  G12D=6.6  G12C=8 |  | |  |  |
