# Supplementary material for: Optimised Pre-Analytical Methods Improve KRAS Mutation Detection in Circulating Tumour DNA (ctDNA) from Patients with Non-Small Cell Lung Cancer (NSCLC)
Source: PLoS One. 2016 Feb 26;11(2):e0150197. doi: 10.1371/journal.pone.0150197 (PMC4769175; doi:10.1371/journal.pone.0150197)
Supplement: S2 Table — (DOCX) [file pone.0150197.s002.docx]

**S2 Table: *KRAS* mutation detection details from various input plasma volumes**

| **Patient** | **Volume of Plasma** | **Control (CT)** | **G12C** | **ΔCT*** |
| --- | --- | --- | --- | --- |
| **Patient 21** | 1ml | 26.66 | Not detected (>45CT) | N/A |
|  | 2ml | 25.72 | Not detected (>45CT) | N/A |
|  | 3ml | 25.17 | Not detected (>45CT) | N/A |
| **Patient** | **Volume of Plasma** | **Control (CT)** | **G12D** | **ΔCT** |
| **Patient 22** | 1ml | 28.55 | Not detected (>45CT) | N/A |
|  | 2ml | 28.31 | Not detected (>45CT) | N/A |
|  | 3ml | 27.29 | Not detected (>45CT) | N/A |
| **Patient** | **Volume of Plasma** | **Control (CT)** | **G12D** | **ΔCT** |
| **Patient 23** | 1ml | 29.51 | 34.91 | 5.4 |
|  | 2ml | 28.02 | 34.41 | 6.39 |
|  | 3ml | 27.5 | 34.63 | 7.13 |
| **Patient** | **Volume of Plasma** | **Control (CT)** | **G12D** | **ΔCT** |
| **Patient 24** | 1ml | 28.96 | Not detected (>45CT) | N/A |
|  | 2ml | 28.13 | Not detected (>45CT) | N/A |
|  | 3ml | 27.25 | 40.34 | 13.09 |
| **Patient** | **Volume of Plasma** | **Control (CT)** | **G12V** | **ΔCT** |
| **Patient 25** | 1ml | 25.01 | 37.4 | 12.39 |
|  | 2ml | 23.54 | 36.37 | 12.83 |
|  | 3ml | 23.16 | 36.33 | 13.17 |
| **Patient** | **Volume of Plasma** | **Control (CT)** | **G12D** | **ΔCT** |
| **Patient 26** | 1ml | 29.56 | Not detected (>45CT) | N/A |
|  | 2ml | 28.44 | Not detected (>45CT) | N/A |
|  | 3ml | 27.86 | 41.42 | 13.56 |
| **Patient** | **Volume of Plasma** | **Control (CT)** | **G12D** | **ΔCT** |
| **Patient 27** | 1ml | 28.6 | 39.81 | 11.21 |
|  | 2ml | 27.61 | 37.49 | 9.88 |
|  | 3ml | 27.23 | 38.43 | 11.2 |

*Qiagen therascreen Delta Ct cutoff

G12V=7.5

G12R=8

G12D=6.6

G12C=8
