# Supplementary material for: Optimised Pre-Analytical Methods Improve KRAS Mutation Detection in Circulating Tumour DNA (ctDNA) from Patients with Non-Small Cell Lung Cancer (NSCLC)
Source: PLoS One. 2016 Feb 26;11(2):e0150197. doi: 10.1371/journal.pone.0150197 (PMC4769175; doi:10.1371/journal.pone.0150197)
Supplement: S3 Table — (DOCX) [file pone.0150197.s003.docx]

**S3 Table: List of commercially available cell free DNA extraction kits**

| **Product Name** | **Vendor** | **Input volume of plasma / serum** | **Considerations** | **References/Location** |
| --- | --- | --- | --- | --- |
| QIAsymphony DSP Virus/Pathogen Kit | QIAGEN | 200 μL – 5 mL* | Automated magnetic bead-based extraction (QIAsymphony)  Allows large sample input | (1) Hilden, Germany |
| QIAamp Circulating Nucleic Acid Kit (CNA) | QIAGEN | 1-5 mL | Column-based method that can be semi-automated on QIAcube. | (2, 3) Hilden, Germany |
| Nucleospin Plasma XS Kit | Macherey-Nagel | <720 μL** | Column-based method. Low elution volume | (4) Duren, Germany |
| FitAmp™ Circulating DNA Quantification Kit | Epigentek | 300 μL | Column-based method.Very low elution volume | (3) Farmingdale, NY,USA |
| Polymer Mediated Enrichment (PME) free-circulating DNA Kit-IPC16 | Analytik Jena | 1-5 mL | Column-based method. Allows large sample input | Jena, Germany |
| ZR Serum DNA Kit | Zymo Research | 100 μL – 10 mL | Bead-based technology -allows large sample input | Irvine, CA, USA |
| Plasma/Serum Cell-Free Circulating DNA Purification kit | Norgen Biotek Corp | 200 – 500 μL |  | (3) Thorold, ON, Canada |
| chemagic Circulating NA kit | Perkin Elmer | 1-4 mL | Automated magnetic extraction (chemagic MSM) | Columbia, MD, USA |
| cobas® DNA Sample Preparation kit | Roche Molecular Systems | 2 mL | This is the tissue kit used by Weber et al 2014– developed for FFPE | (5) Pleasanton, CA, USA |

* Modified 1000 protocol enables use of up to 5mL, ** multiple loading steps

References

1. Nocon A, Horlitz M, Wolf A, Scholtysik P: Automated large-volume extraction of circulating, cell-free DNA to improve the sensitivity of tumor biomarker detection. Journal of Clinical Oncology 2012, 30:S30.

2. Devonshire AS, Whale AS, Gutteridge A, Jones G, Cowen S, Foy CA, Huggett JF: Towards standardisation of cell-free DNA measurement in plasma: controls for extraction efficiency, fragment size bias and quantification. Analytical and Bioanalytical Chemistry 2014, 406:6499-6512.

3. Page K, Guttery DS, Zahra N, Primrose L, Elshaw SR, Pringle JH, Blighe K, Marchese SD, Hills A, Woodley L, Stebbing J, Coombes RC, Shaw JA: Influence of Plasma Processing on Recovery and Analysis of Circulating Nucleic Acids. PLoS ONE 2013, 8:e77963.

4. Hufnagl C, Stöcher M, Moik M, Geisberger R, Greil R: A modified Phenol-chloroform extraction method for isolating circulating cell free DNA of tumor patients. Journal of Nucleic Acids Investigation 2013, 4:e1.

5. Weber B, Meldgaard P, Hager H, Wu L, Wei W, Tsai J, Khalil A, Nexo E, Sorensen B: Detection of EGFR mutations in plasma and biopsies from non-small cell lung cancer patients by allele-specific PCR assays. BMC Cancer 2014, 14:294.
